# A Sexually Dimorphic Corolla Appendage Affects Pollen Removal and Floral Longevity in Gynodioecious *Cyananthus delavayi* (Campanulaceae)

**DOI:** 10.1371/journal.pone.0117149

**Published:** 2015-01-20

**Authors:** Yang Niu, Zhi-Qiang Zhang, Chang-Qiu Liu, Zhi-Min Li, Hang Sun

**Affiliations:** 1 Key Laboratory for Plant Diversity and Biogeography of East Asia, Kunming Institute of Botany, Chinese Academy of Sciences, Kunming, Yunnan, China; 2 School of Life Sciences, Yunnan Normal University, Kunming, Yunnan, China; McGill University, CANADA

## Abstract

The floral traits of bisexual flowers may evolve in response to selection on both male and female functions, but the relative importance of selection associated with each of these two aspects is poorly resolved. Sexually dimorphic traits in plants with unisexual flowers may reflect gender-specific selection, providing opportunities for gaining an increased understanding of the evolution of specific floral traits. We examined sexually dimorphic patterns of floral traits in perfect and female flowers of the gynodioecious species *Cyananthus delavayi*. A special corolla appendage, the throat hair, was investigated experimentally to examine its influences on male and female function. We found that perfect flowers have larger corollas and much longer throat hairs than female flowers, while female ones have much exerted stigmas. The presence of throat hairs prolonged the duration of pollen presentation by restricting the amount of pollen removed by pollen-collecting bees during each visit. Floral longevity was negatively related to the rate of pollen removal. When pollen removal rate was limited in perfect flowers, the duration of the female phases diminished with the increased male phase duration. There was a weak negative correlation between throat hair length and seed number per fruit in female flowers, but this correlation was not significant in perfect flowers. These results suggest that throat hairs may enhance male function in terms of prolonged pollen presentation. However, throat hairs have no obvious effect on female function in terms of seed number per fruit. The marked sexual dimorphism of this corolla appendage in *C. delavayi* is likely to have evolved and been maintained by gender-specific selection.

## Introduction

Floral characters in hermaphroditic plants may be selected via both male and female sexual functions (reviewed in [1]). However, two important links to achieve male and female fitness, pollen removal and receipt, are conceived as separate processes. Floral traits that promote the deposition of pollen on stigmas may not be optimal for the removal of pollen from anthers [2]. For example, height in *Impatiens pallida* was found to be under positive selection for pollen receipt, but negative selection for pollen export [3]. There is therefore a potential conflict between the two sexual functions [4]. This conflict may also arise because of the difference in reproductive strategies between the sexual functions. Given that the proportion of pollen deposited on conspecific stigmas diminishes as the amount of pollen removed increases in a single visit, a plant can generally maximize pollen dispersal by limiting the amount of pollen removed in a single visit [2,5,6]. Pollen packaging (package pollen in inflorescences, individual flowers, stamens and anther sacs) and dispensing (e.g., secondary pollen presentation and special ways of anther dehiscence) have been suggested to be the common mechanisms that deliver pollen to pollinators among multiple visits [2,5,7,8]. However, mechanisms that fulfill the requirement of restricting pollen removal often result in reduced attractiveness to pollinators, and this may not be optimal for pollen deposition [5].

This conflict of functions is avoided or greatly reduced in species with sexual functions separated temporally (herkogamy) and spatially (dichogamy). However, in bisexual flowers, herkogamy may involve an additional conflict with pollination accuracy [9] (see [10,11]). And within-flower dichogamy may also induce a conflict in terms of the time division between sexual phases that share the limited floral lifespan. In systems with unisexual flowers, strong disruptive selection between sexual functions can promote the evolution of phenotypes (including ancillary floral structures) that are closer to the fitness optimum for male and female function [12], resulting in sexual dimorphism.

Sexual dimorphism in floral traits and secondary sexual characteristics are not uncommon in plants, with perianth size, nectar production, and floral scent among other traits differing between morphs (reviewed in [13–15]). These dimorphisms provide clues leading to a better understanding of the effects of gender-specific selection on floral traits. Gynodioecy includes hermaphrodites and male-sterile (female) individuals in the same population. In addition to the dimorphism in male and female organs, dimorphism of secondary floral traits (e.g., perianth size and nectar production) are often present in gynodioecious taxa [14]. In a gynodioecious system, females often set more seeds than hermaphrodites and hermaphrodites function mainly as pollen donors (reviewed in [16,17]). It is therefore possible that some floral traits in hermaphrodites and females have been selected via the male and female functions, respectively.

**Figure 1:**
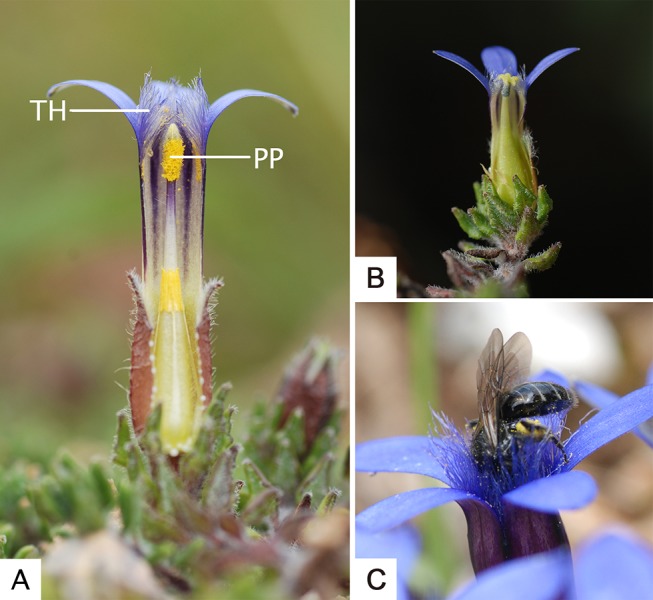
Floral traits of *Cyananthus delavayi* and its pollinator in the natural population. A: Dissected view of perfect flowers, showing long throat hair (TH) and pollen presentation area (PP); B: Dissected view of female flowers, showing short throat hair and exerted stigma; C: Pollen-collecting *Halictus* sp. crawling into a perfect flower in the male phase. A and B are modified from [20]. All pictures are photographed by Yang Niu.

*Cyananthus* Wall. ex Benth. (Campanulaceae) is a genus endemic to the east Himalayas-Hengduan Mountains region [18]. *Cyananthus* comprises three clades (sections), and one of these (Sect. Stenolobe (Franchet) Y.S. Lian) is dominated by gynodioecious sexual systems [19]. Many species that belong to this section have dense hairs covering the corolla throat (Fig. 1); we refer to this special corolla appendage as throat hair. According to our observations, this structure may influence pollen export by impeding the pathway of pollinators while they are accessing pollen. This may restrict pollen removal, facilitating pollen dispensing when the number of visits is sufficient. However, the presence of throat hairs may reduce female function at the same time by hindering pollen deposition. Similar constricted floral parts had already been documented in other taxa, such as *Campanula zoysii* and *Primula primulina* [16] (and *Primula bella*, personal observation), but the adaptive significance of such structures has seldom been investigated. In preliminary studies of *Cyananthus delavayi* Franch., a gynodioecious perennial, we found that throat hair is well-developed in perfect flowers but underdeveloped in female flowers, representing a clear sexual dimorphism (Fig. 1A and B). Therefore, we hypothesize that gender-specific selection may contribute to the observed dimorphism of this trait. We therefore predict that, (1) floral traits of perfect and female flowers should show significant sexual dimorphism, promoting male and female functions respectively; (2) The presence of throat hairs may limit the rate of pollen export and subsequently increases male fitness, despite a possible cost to female fitness. To test these predictions, we first analyzed the sexually dimorphic pattern of several floral traits, including throat hair. Hair removal was then used to examine the effects of throat hair on male and female function in terms of pollen removal rate and floral longevity. The correlation between throat hair length and seed number per fruit was also estimated in both sexual morphs.

## Materials and Methods

### Study species and locality

*Cyananthus delavayi* (Campanulaceae) is a perennial herb with a gynodioecious sexual system [20]. The female and hermaphrodite morphs coexist in all the populations we observed (female frequency is 53% in the population we studied). The genetic basis of sex determination and expression for this species is unknown. Hermaphrodites produce perfect flowers with a secondary pollen presentation mechanism. The perfect flowers are self-compatible, but both auto- and facilitated-selfing within flowers are avoided by complete protandry. During observations from 2007 to 2013, flowers were visited by pollen-collecting halictid bees, nectar-feeding bumble bees and pollen-feeding hoverflies. Of these, halictid bees were the most abundant visitors, accounting for over half of all visitations [21]. They showed strong preference for pollen-producing flowers, i.e. perfect flowers in the male-phase. In a previous study, we found that pollen deposition could induce permanent floral closure [20], and in the current study we found that pollen removal could also reduce floral longevity significantly. The throat hairs of *C*. *delavayi* were obvious in perfect flowers but less evident in female flowers (Fig. 1A and B). Female individuals produced much higher numbers of flowers and produced many more seeds than the hermaphrodites [21]. The seeds were rarely consumed by predators in this species (see [22]).

Our field study site (27°54′N, 99°38’E, elevation 3355 m a.s.l.) is located in the Shangri-la Alpine Botanical Garden (Shangri-la County, northwest of Yunnan province). This garden is a natural preserve where the subalpine flora is protected from grazing (see more details in [20]).

### Sexual dimorphism in floral traits

In order to investigate whether *C*. *delavayi* exhibits sexual dimorphism in floral traits between hermaphrodites and females, in 2012 the following morphological parameters were measured in the field using a digital caliper (± 0.01 mm): corolla diameter, throat diameter, corolla tube length, style length, stigma diameter and throat hair length. Style length divided by corolla tube length was used to calculate relative stigma position (only the latter data are presented), with a higher value indicating a more exerted stigma and values near 1.0 indicating anther-stigma overlap. Since some floral traits may develop and show different patterns (which may relate to sexual functions) as anthesis proceeds, we divided flowers into three categories according to their gender phase or age. For perfect flowers, three phases were defined: the male phase (day 1, see detailed definition in the next section), the neutral phase (days 2 or 3) and the female phase (days 4–5). For female flowers, where gender phase did not apply, the corresponding ages were day 1, day 3 and day 5. Three flowers (each representing one of the three phases or ages) from each plant were selected. In total, 30 hermaphrodites (30 × 3 flowers) and 34 females (34 × 3 flowers) were measured. Data were compared between sexes within the same period, using independent samples *t*-tests, and among phases within each sex, using one-way ANOVA followed by Tukey’s test. We also used the mean floral trait values (averaged from the three phases/ages) for each sex to calculate an index of sexual dimorphism (SD) [23–25]:

SD = [mean trait value in the larger sex morph/ mean trait value in the smaller sex morph] – 1

### The effects of pollen removal and throat hair manipulation on floral longevity

**Figure 2:**
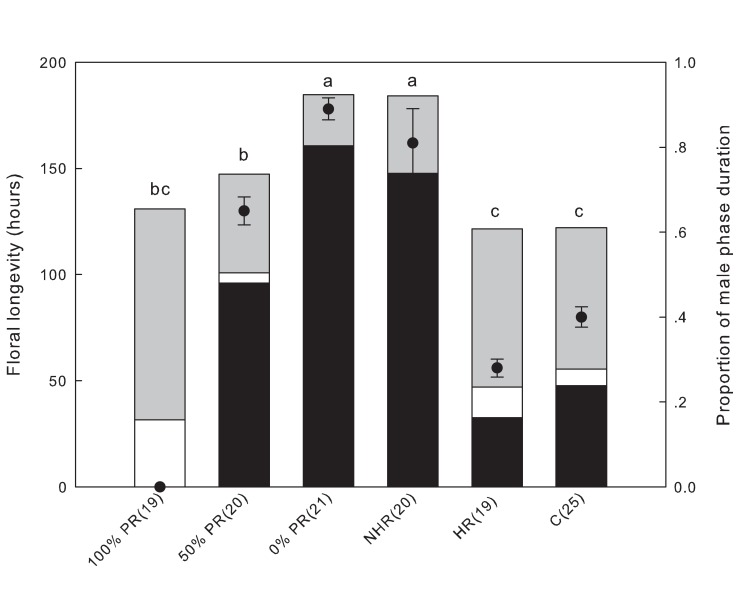
Pollen removal and throat hair manipulation had significant effects on floral longevity in perfect flowers. Black, white and gray bars represent male, neutral and female phases, respectively. Solid circles represent the proportion of male phase duration (male phase divided by total floral longevity). Along the X-axis, 100% PR, 50% PR and 0% PR indicate pollen completely removed, 50% pollen removed and no pollen removed, respectively (these groups had pollinators excluded); NHR indicates the hair-removal group covered by netting; HR and C indicate hair-removal and natural control groups open to pollinators, respectively. Different lower-case letters above the bars indicate significant differences in total floral longevity at the 0.01 level. Different capital letters on the black bars indicate significant differences in male phase duration (and also in the proportion of male phase) at the 0.01 level. Numbers in brackets are sample sizes. Bars are standard errors.

As we speculate that throat hairs may influence floral longevity through their effects on pollen removal, we first examined whether floral longevity is influenced by pollen removal, and then investigated the effects of throat hairs on pollen removal. In 2009, 125 perfect flowers (from 20 plants) were randomly assigned to six treatments (see Fig. 2 for sample sizes): 1) no pollen removal (0% PR); 2) ∼50% pollen removed (50% PR); 3) ∼100% pollen removed (100% PR); 4) netted flowers with hair removed (NHR); 5) hair removed but natural pollination allowed (HR); and 6) control (C). Pollinators were excluded from the first four treatments, while the latter two were open to pollinators. Pollinator exclusion was achieved by placing a nylon net over part of the plant. Pollen grains were removed with a fine paintbrush dampened with distilled water. Throat hair removal was conducted carefully with fine scissors, and this treatment did not affect other conditions such as the number of pollen grains (24039 ± 704 vs. 24140 ± 688, *t* = −0.103; df = 38; *P* = 0.919) and the floral tube structure. All of the treatments were applied on the first day of anthesis.

The gender phase (male, neutral, female, closed or withered) of each flower was recorded twice every day at 1030–1130 h and 1630–1730 h. Phase durations were calculated in hours. The male phase of *C*. *delavayi* is defined as the period from initial opening of the corolla to complete pollen removal; from this time to when the stigmatic lobe unfolds is defined as the neutral phase; and the female phase is defined as the period from stigmatic lobe expansion until flower wilting or permanent closure. Note that, on this basis, there is no male phase in the 100% PR group (this group was therefore not included in the comparison of male phase duration). There were no rainy days during the experimental period. The total floral longevity and the proportion of male phase among the treatments were analyzed by one-way ANOVA.

### The effects of throat hair on pollen removal

In order to test whether the throat hair has any effect on pollen removal, we compared the amount of pollen removed after a single visit by a halictid bee (*Halictus* sp., Fig. 1C) when throat hair was present or absent. We selected halictid bees because they were the most abundant visitors observed over a period of several years. In 2009, pollinators were excluded from fifteen hermaphroditic plants before anthesis; of these, the throat hair of 50 randomly-selected flowers was removed at the onset of anthesis (HR), other flowers in the same condition was left intact as a control (C), and both groups were left open to visitors. In total, 34 flowers from the HR group and 30 from the control group were harvested after they had received a single visit by a halictid bee. The pollen presentation areas of these flowers were placed into individual 1.5-mL Eppendorf tubes containing 70% ethanol before pollen counting. The tubes were concussed to dislodge all pollen, and the number of pollen grains remaining in each flower was determined under a microscope. We also estimated the total amount of pollen contained in a flower from 20 perfect flowers collected before anthesis. Three 10 μL sub-samples were taken from each sample and the number of pollen grains in each sub-sample was counted under a microscope. The average number of pollen grains per flower was then estimated. Finally, the number of pollen grains removed during a single bee visit was calculated by subtracting the number of pollen grains remaining in the flower from the total number of pollen grains. This value was used to calculate the proportion of pollen removed. An independent samples *t*-test was used to analyze the numerical data (with log-transformed). A Mann-Whitney U test was used to analyze data expressed as proportions.

### The effects of throat hair on female reproductive success

Throat hair may have an effect on female reproductive success through its influence on pollen deposition, and thus this effect may ultimately be reflected in the seed number per fruit. Although throat hair was well-developed in perfect flowers and relatively underdeveloped in female flowers, they both showed some variation in length among individuals. Using this variation, we examined whether throat hair has any effect on seed number per fruit. To do this, we tagged 20 individuals in 2012, then measured throat hair length (averaged from two to three flowers) and counted the number of seeds per fruit (expressed as an average across all fruits for each individual plant) when fruit had matured. As a large number of samples were lost due to the construction of the garden, we repeated this experiment in 2013. In total, we obtained data from 17 hermaphrodites and 24 females for both throat hair length and seed number data; and these data, obtained from two years, were pooled in the analysis. Pearson correlation was used to analyze the relationship between throat hair length and number of seeds per fruit. All the statistical analyses referred to above were conducted using SPSS (version 13.0, SPSS Inc., Chicago, IL, USA).

## Results

### Sexual dimorphism in floral traits

Results of the one-way ANOVA within each sex showed that in both perfect and female flowers, with the exception of stigma position, the measured values of floral traits increased significantly as anthesis proceeded (S1 Table). For each phase, perfect flowers exhibited larger corollas and much longer throat hair than female flowers, but female flowers had more exerted stigmas (Table 1). There was no significant difference in stigma diameter (Table 1). Of the floral traits assessed, throat hair was associated with the highest index of sexual dimorphism (SD = 0.63, Table 1): female flowers had much shorter throat hair than perfect flowers. Fig. 1A and B show the pollen presentation area covered by well-developed throat hair in perfect flowers (male phase), while in female flowers the stigma is not obviously covered by throat hair.

**Table 1:** The sexually dimorphic pattern of floral traits (mean ± SE) in *Cyananthus delavayi*.

Phase/Age	Sex	Corolla diameter (mm)	Throat diameter (mm)	Stigma position	Stigma diameter (mm)	throat hair length (mm)
Male/Day 1	H	13.14 ± 0.33 ^**^	3.50 ± 0.06^**^	1.02 ± 0.01^**^	-	3.59 ± 0.06^**^
	F	11.87 ± 0.26	3.19 ± 0.05	1.12 ± 0.01	-	2.16 ± 0.05
Neutral/Day 3	H	17.22 ± 0.42 ^**^	3.98 ± 0.07^**^	1.02 ± 0.01 ^**^	-	3.96 ± 0.06^**^
	F	13.94 ± 0.25	3.40 ± 0.06	1.10 ± 0.01	-	2.42 ± 0.07
Female/Day 5	H	21.74 ± 0.46^**^	4.30 ± 0.07^**^	1.04 ± 0.01^**^	2.15 ± 0.04^NS^	4.00 ± 0.07^**^
	F	16.07 ± 0.31	3.64 ± 0.05	1.11 ± 0.01	2.17 ± 0.04	2.51 ± 0.08
SD		0.24	0.15	0.09	-	0.63

The data were compared between flowers from hermaphroditic (H, *N* = 30) and female (F, *N* = 34) individuals during three gender phases (male, neutral and female in perfect flowers and correspondingly days 1, 3 and 5 in female flowers), using an independent samples *t*-test. “Stigma position” was calculated by dividing style length by tube length, a larger value indicates a more exerted stigma. “SD” indicates the index of sexual dimorphism (see details in the main text). The superscripts “NS” and “**” indicate non-significant and significant difference at the 0.01 level, respectively.

### The effects of pollen removal and throat hair manipulation on floral longevity

Pollen removal and manipulation of throat hair had significant effects on floral longevity. All the pollinator-excluded groups had significantly greater total longevity, a longer male phase and a higher proportion of male phase (with the exception of 100% PR, which had no male phase according to our definition) than the groups open to pollinators (Fig. 2; for total floral longevity *F*_*5*, *118*_ = 31.02, *P* < 0.001; for male phase duration *F*_*4*, *100*_ = 189.77, *P* < 0.001; for proportion of male phase *F*_*4*, *100*_ = 83.67, *P* < 0.001). In groups covered by netting, both total floral longevity and male phase duration increased significantly with decreasing pollen removal, but the difference between 0% PR and NHR (i.e., the two groups with no pollen removal) was not significant (Fig. 2). In groups open to pollinators, male phase duration and male phase proportion in HR were significantly lower than in the control, but the difference in total floral longevity was not significant. In addition, it is noteworthy that neutral and female phase durations were prolonged in HR groups, as well as other groups, when pollen was removed (i.e., 100% PR and 50% PR), demonstrating a shift between the two gender phases within the limited floral lifespan. We also noticed that the female phase never occurred in about one third of the flowers with no pollen removed (0% PR and NHR).

### The effects of throat hair on pollen removal

**Figure 3:**
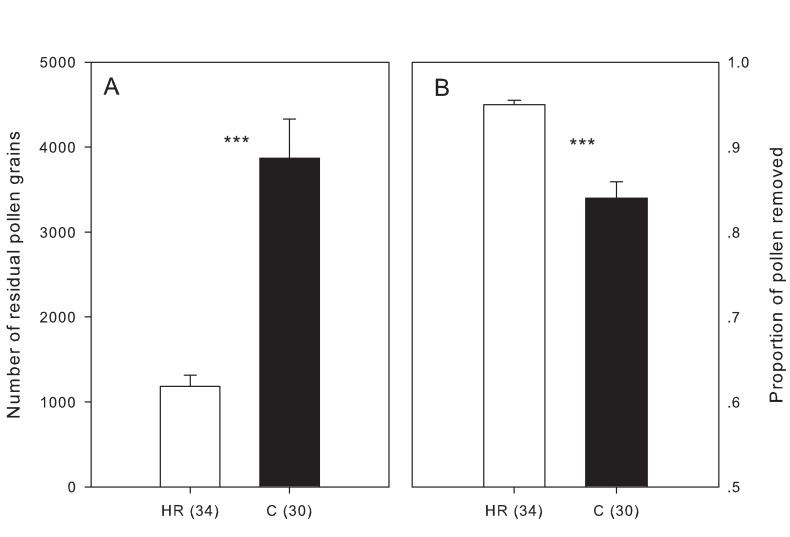
The presence of throat hair increased the number of pollen grains remaining in the flowers (A) and decreased the proportion of pollen removed (B) in single visits by halictid bees. “HR” and “C” indicate hair-removal (white bar) and control groups (black bar), respectively. Significant differences at the 0.001 level are indicated by “***”. Numbers in brackets are sample sizes. Bars indicate standard errors.

The total number of pollen grains in perfect flowers of *C*. *delavayi* was found to be 24140 ± 154 (mean ± SE, *N* = 20). Our results showed that throat hair excision had significant effects on pollen removal by halictid bees. After a single visit, a much smaller (*t* = −6.682, *df* = 62, *P* < 0.001) number of pollen grains remained in HR flowers than in intact flowers. The estimated ratio of pollen removed was significantly higher for flowers in the HR group than for flowers in the control group (Fig. 3, *Z* = 5.025, *P* < 0.001).

### The correlation between throat hair length and seed number per fruit

**Figure 4:**
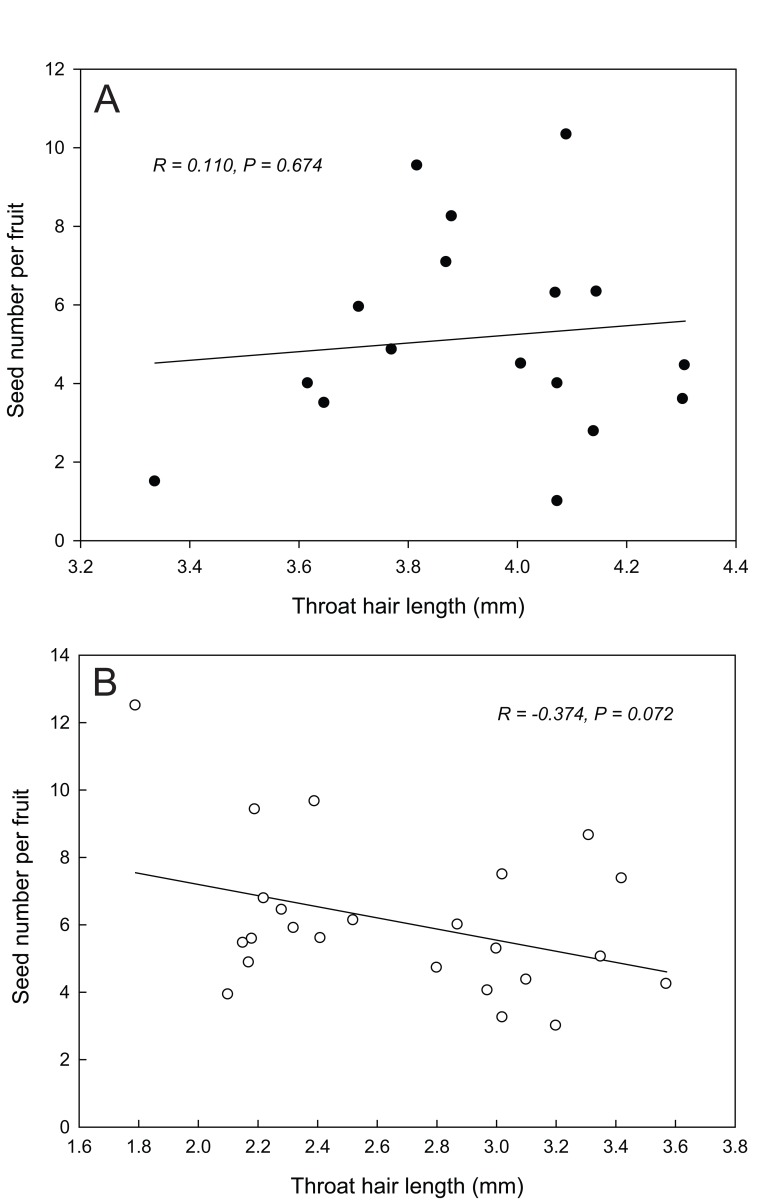
The Pearson correlation between throat hair length and seed number per fruit. The correlation was not significant in perfect flowers (A) but marginal significant in female flowers (B).

For hermaphroditic individuals, there was no significant correlation between seed number per fruit and throat hair length of perfect flowers (Fig. 4A; *R* = 0.038, *P* = 0.886, *N* = 17). However, for female flowers this correlation was marginally significant (Fig. 4B; *R* = −0.374, *P* = 0.072, *N* = 24), indicating a slight trend that throat hair negatively related to seed number per fruit.

## Discussion

### Sexual dimorphism in floral traits

We found that the floral traits measured were generally larger in perfect flowers than female flowers, a phenomenon consistent with Eckhart’s finding in gynodioecious species more broadly [14]. This pattern may be explained by the “development-correlation” hypothesis, i.e. that there is a developmental correlation between the development of stamens and other floral traits [26,27]. However, we noticed that not all floral traits exhibit the same pattern in *C*. *delavayi*; for example, there was no significant difference in stigma diameter between sexes. In addition, although the length of corolla tube and style were greater in perfect flowers, when the relative positions of these two traits were considered, female flowers showed more exerted stigmas than perfect flowers (Table 1). Wide and exerted stigmas are considered an adaptation to maximize pollen receipt. Furthermore, among the traits we measured, throat hair showed the highest SD value, indicating a disproportionately high level of sexual dimorphism relative to other traits. Alternative to the developmental-correlation hypothesis, divergence in throat hair is more likely to represent differential selection on sexual function for pollen export and pollen receipt.

### The effects of throat hair on male and female functions

Floral longevity affects plants’ reproductive success through its effects on pollen presentation and receipt [28]. However, maintaining flowers in a particular functional state consumes resources that could otherwise be allocated to other processes [29,30]. It is thus reasonable to predict that once pollination and/or fertilization is complete, the life of a flower should finish as soon as possible in order to minimize the costs of flower maintenance [31]. Empirical studies do, indeed, show that floral longevity is often decreased after successful pollination (reviewed in [31]). In *C*. *delavayi*, the female phase was dramatically shortened once flowers had received sufficient pollen [20], and the duration of the male phase was negatively related to the rate of pollen removal (Fig. 2). We further showed that the duration of pollen presentation was prolonged when throat hair was present, because the pollen removal rate was restricted by the dense hair when natural pollination took place. Under natural conditions, all the flowers we observed had exported their pollen completely within two days (Fig. 2), and thus the restriction on pollen removal did not decrease the quantity of pollen export in total, but means a flower requires more visits to export the same quantity of pollen when throat hair was present. Pollen-collecting halictid bees showed a strong preference for male phase perfect flowers [21]. This discrimination in visitation combined with a high rate of pollen removal often made these bees low-efficiency pollinators [32,33] (see also [10] for similar findings in the same area). The restriction of pollen removal by of throat hair allows pollen to be distributed amongst a greater variety of recipients, decreasing pollen wastage and reducing sibling competition, and thus enhancing male function [5,34,35]. We acknowledge that our results are based on data pertaining to the most common pollinators, which mainly collect pollen, but the results may also apply to nectar-feeding pollinators. We did find that the nectar volume of male phase flowers was much lower than female phase flowers (0.28 ± 0.04 vs. 1.08 ± 0.07 μL, *t* = 10.588, *df* = 61, *P* < 0.001, [21]). This pattern reduced the attractiveness of male phase flowers, and has been considered a means to restrict pollen removal by nectar-feeding pollinators [36]. Collectively, these results indicate that throat hair has positive effects on male function. On the other hand, we did not find any positive effects of throat hair on female function in perfect flowers (Fig. 4A), in terms of seed number per fruit. While, our results showed a trend (*P* = 0.07) that seed number per fruit in female flowers negatively related to throat hair length (Fig. 4B), possibly because throat hair hinders pollen deposition. To understand these different results between sexual morphs, we assume that it is possible that throat hair only covered the corolla throat in male phase of perfect flowers, and the pathway to the stigma was unobstructed in the female phase because of the much expanded corolla throat in older flowers (S1 Table). Although the corolla throat also expanded in female flowers as the anthesis proceeded, it may already have an effect in the early days of anthesis. In addition, the corolla throat in female flowers was narrower than in perfect ones in general (Table 1), which may have stronger effects on pollen deposition when throat hair is present. As well as the physical interference, our results suggest that the presence of throat hair prolongs the relative duration of the male phase at the cost of the female phase, potentially reducing female fitness (see the following section). Collectively, these results suggest that the function of throat hair is male-biased.

### Sexual interference in *Cyananthus delavayi*

Potential sexual interference in *C*. *delavayi* may arise from the different effects of throat hair on pollen dispersal and deposition. It has been suggested that restricting pollen removal tends to reduce the foraging efficiency of pollen-collectors and limit the production of nectar for nectar foragers, and both of these factors may decrease the flower’s attractiveness and compromise the efficiency of pollen deposition [5]. In the current study, the presence of throat hair enhanced male fitness by prolonging pollen presentation duration but possibly diminished female fitness through impeding pollen deposition. The sexual dimorphism of the throat hair in *C*. *delavayi* may, therefore, be regarded as an evolutionary solution that greatly reduces this interference. It is true that the weak negative correlation between throat hair length and seed number per fruit may result from an internal correlation that such plants with shorter throat hair devote more resource to seed production than those with longer hair. Nevertheless, both these possibilities would facilitate the maintenance of sexual dimorphism of this floral trait. However, as we observed, throat hair may not be eliminated completely in females because of potential genetic correlation.

The separation of sexual functions in time (especially protandry, the most widespread form), is believed to be an efficient mechanism for avoiding sexual interference [3,9,37,38]. However, in this study we showed that even in dichogamous flowers interference can still take place between sexual functions which operate successively: an extension of one gender phase may be at the cost of the duration of the other. In the protandrous perfect flowers of *C*. *delavayi*, male and female phases occupied almost equal shares of the total floral life when the flower was naturally pollinated (Fig. 2, group C). However, when we limited the rate of pollen removal, the male phase was greatly prolonged at the expense of the female phase (Fig. 2, groups 50% PR, 0% PR and NHR). The female phase never occurred in about one third of the flowers from which no pollen was removed (0% PR and NHR), although the total floral longevity was not altered. This compensatory relationship has only been reported in a few other protandrous flowers, with similar secondary pollen presentation behavior [39,40]. In the hermaphroditic plant *Erythronium japonicum*, researchers also concluded that a minimum floral longevity may exist for male functions rather than female function [41]. Here we suppose there are two possible reasons why male function took priority in *C*. *delavayi*. First, the male function has an inherent advantage in protandrous flowers because it precedes the female function [9], especially when the duration of the male phase depends on pollen removal. Secondly, perfect flowers function mainly as fathers in the context of gynodioecy [16,17]. In such a scenario, especially when female frequency is high, selection will favor the floral traits that enhance the male function of the hermaphrodites [16,42].

## Supporting Information

S1 Table The development of floral traits in hermaphrodites (H, *N* = 30) and females (F, *N* = 34) as anthesis proceeded of *Cyananthus delavayi*. “Stigma position” was calculated by dividing style length by tube length, and a larger value indicates a more exerted stigma. Data were analyzed by one-way ANOVA (followed by Tukey test) for each sex, with phase or age as the factor. Different lowercase letters indicates significant difference among phases (for perfect flowers: male, neutral and female phase) or ages (for female flowers, day1, 3 and 5, correspondingly) at 0.05 level.
(DOC)
