# Supplementary material for: A Sexually Dimorphic Corolla Appendage Affects Pollen Removal and Floral Longevity in Gynodioecious Cyananthus delavayi (Campanulaceae)
Source: PLoS One. 2015 Jan 20;10(1):e0117149. doi: 10.1371/journal.pone.0117149 (PMC4300179; doi:10.1371/journal.pone.0117149)
Supplement: S1 Table — “Stigma position” was calculated by dividing style length by tube length, and a larger value indicates a more exerted stigma. Data were analyzed by one-way ANOVA (followed by Tukey test) for each sex, with phase or age as the factor. Different lowercase letters indicates significant difference among phases (for perfect flowers: male, neutral and female phase) or ages (for female flowers, day1, 3 and 5, correspondingly) at 0.05 level. (DOC) [file pone.0117149.s001.doc]

**Table S1 The development of floral traits in hermaphrodites (H, *N* = 30) and females (F, *N* = 34) as anthesis proceeded of *Cyananthus delavayi*.** “Stigma position” was calculated by dividing style length by tube length, and a larger value indicates a more exerted stigma. Data were analyzed by one-way ANOVA (followed by Tukey test) for each sex, with phase or age as the factor. Different lowercase letters indicates significant difference among phases (for perfect flowers: male, neutral and female phase) or ages (for female flowers, day1, 3 and 5, correspondingly) at 0.05 level.

| **Sex** | **Phase/age** | **Corolla diameter**  **(mm)** | **Throat diameter**  **(mm)** | **Stigma diameter**  **(mm)** | **Stigma position** | **Throat hair length**  **(mm)** |
| --- | --- | --- | --- | --- | --- | --- |
| **H** | male | 13.14 ± 0.33^a^ | 3.51 ± 0.06^a^ | - | 1.02 ± 0.01^a^ | 3.58 ± 0.06^a^ |
|  | neutral | 17.22 ± 0.42^b^ | 3.98 ± 0.07^b^ | - | 1.02 ± 0.01^a^ | 3.96 ± 0.06^b^ |
|  | female | 21.73 ± 0.46^c^ | 4.29 ± 0.07^c^ | 2.14 ± 0.03 | 1.04 ± 0.01^a^ | 4.00 ± 0.07^b^ |
|  | *F* | 109.888 | 33.617 | - | 0.806 | 14.159 |
|  | *P* | **< 0.001** | **< 0.001** | - | 0.450 | **< 0.001** |
|  |  |  |  |  |  |  |
| **F** | day1 | 11.87 ± 0.26^a^ | 3.19 ± 0.05^a^ | 2.00 ± 0.04^a^ | 1.12 ± 0.01^a^ | 2.16 ± 0.05^a^ |
|  | day3 | 13.94 ± 0.25^b^ | 3.41 ± 0.06^b^ | 2.09 ± 0.03^ab^ | 1.11 ± 0.01^a^ | 2.42 ± 0.08^b^ |
|  | day5 | 16.07 ± 0.31^c^ | 3.64 ± 0.05^c^ | 2.17 ± 0.04^b^ | 1.11 ± 0.01^a^ | 2.51 ± 0.08^b^ |
|  | *F* | 57.752 | 18.825 | 5.241 | 0.976 | 6.682 |
|  | *P* | **< 0.001** | **< 0.001** | **0.007** | 0.380 | **0.002** |
